# Uric acid reduces the expression of aquaporins in renal collecting ducts to increase urine output in hyperuricemia

**DOI:** 10.3389/fphys.2025.1504328

**Published:** 2025-04-09

**Authors:** Xiaohui Cui, Rongfang Qiao, Bing Wang, Yitong Hu, Guoying Sun, Wenjuan Hu, Zhilin Luan, Huiwen Ren, Hu Xu, Youfei Guan, Xiaoyan Zhang

**Affiliations:** ^1^ Advanced Institute for Medical Sciences, Dalian Medical University, Dalian, China; ^2^ Department of Endocrinology and Metabolism, The Central hospital of Dalian University of Technology, Dalian, China; ^3^ Kidney Health Institute, East China Normal University, Shanghai, China

**Keywords:** hyperuricemia, uric acid, aquaporin, NF-κB, collecting ducts

## Abstract

**Background:**

Hyperuricemia (HUA) has attracted wide attention due to its close relationship with gout, hypertension, hypertriglyceridemia, obesity, atherosclerotic heart disease, type 2 diabetes and chronic kidney disease. Clinical observations suggest that people with high levels of serum uric acid (sUA) exhibits impaired urine concentration. We speculate that UA may regulate the expression of AQPs through inflammatory pathways, resulting in impaired renal urine concentration.

**Methods and results:**

We revealed that patients and mice with HUA had a polyuria phenotype and found that the expression of aquaporin 2 (AQP2), AQP3 and AQP4 were significantly reduced in the kidneys of mice with HUA. Similarly, uric acid (UA) treatment markedly suppressed the expression of AQP2, AQP3 and AQP4 in cultured inner medullary collecting duct cells (IMCDs). We observed an increased expression of NF-κB in the kidneys of mice with HUA and in the IMCD cells treated with UA. Blockade of NF-κB by its inhibitor Bay 11-7082 dramatically attenuated UA-suppressed expression of AQP2, AQP3 and AQP4. Furthermore, the luciferase reporter, CHIP and EMSA assays showed that NF-κB can directly bind to the promoter regions of AQP2, AQP3 and AQP4 genes to suppress their transcription.

**Conclusion:**

Our findings demonstrate that UA reduces the expression of AQP2, AQP3 and AQP4 in an NFκB-dependent manner, which contributes to the polyuria phenotype in the subjects with HUA.

## 1 Introduction

Uric acid (UA) is the end-product of purine catabolism, which is synthesized mainly in the liver. The adult human body produces about 700 mg of UA each day (El Ridi and Tallima, 2017). The kidney is the main organ for UA disposal, with about 70% of UA excreted in the urine (Maesaka and Fishbane, 1998), and the remaining 30% excreted from the intestine and biliary tract (Sorensen, 1965). When renal function is impaired, the intestine will become the dominant organ of UA excretion. The serum uric acid (sUA) level is governed by the balance of production and excretion. Under a normal purine diet, people with fasting sUA levels greater or equal to 420 μmol/L (7 mg/dL) measured twice on different days can be diagnosed as hyperuricemia (HUA). With rapid improvement of living standards and the changes of life style, the prevalence and incidence of HUA have been constantly increasing especially in young population due to the excessive intake of purines and sugars.

Excessive accumulation of UA leads to HUA and urate crystal deposition in tissues including joints and kidneys. A large body of evidence indicates that HUA is a risk factor of gout, renal diseases, cardiovascular diseases, hypertension, and metabolic syndrome (Bartáková et al., 2016; Yanai et al., 2021; Johnson et al., 2018a; Mazzali et al., 2002; Feig and Johnson, 2003). Studies have shown that the reduction of sUA levels prevents acute kidney injury and improves chronic kidney disease (Ogino et al., 2010). However, clinical observation has also reported that people with higher levels of sUA appear to have increased 24-h urine output (Johnson et al., 2018b). Similarly, in a rat HUA model with the uric acid oxidase (UOX) gene deficiency, the 24-h urine volume was significantly increased compared with the wild-type rats (Gao et al., 2022)^.^ These findings demonstrate that patients and animals with HUA seem to have a defect in urine concentration, leading to a polyuria phenotype, with the underlying mechanism incompletely elucidated.

The kidneys play a critical role in maintaining body water homeostasis via adjusting urine concentration and dilution. The reabsorption of water in the kidney is mainly regulated by the water transport protein family, named aquaporins (AQPs), which are localized in the epithelial cells of certain renal tubules. Within the kidney, AQP1 is expressed at the apical membrane and basolateral membrane of proximal tubules and thin descending limb of Henle (tDLH), responsible for constitutive water reabsorption (Pallone et al., 1997). AQP2 is expressed at the apical membrane, while AQP3 and AQP4 are expressed at the basolateral membrane of the collecting ducts. The water transport in the collecting ducts is of great significance to the regulatory reabsorption of water (Rojek et al., 2006; Yang and Verkman, 1997; Ma et al., 2000). Dysfunction and dysregulation of these AQPs result in various water balance disorders, such as nephrogenic diabetes insipidus, which is characterized by inability to concentrate urine, resulting in polyuria and polydipsia (Nielsen et al., 2002; Chou et al., 1998).

A large body of evidence indicates that the expression of renal AQPs is regulated by a variety of factors including inflammation-related factors. A series of transcriptome changes in rat renal collecting duct cells during the early stage of ureteral obstruction showed that upregulation of inflammatory pathways is associated with AQP2 protein loss in the kidney (Sung et al., 2022). In the kidneys of rats with ischemia reperfusion injury, activation of NF-κB signaling pathway increases the expression of IL-1β and inhibits the expression of AQP2 (Han et al., 2021). It has been reported that knocking down the expression of NF-κB in rat collecting duct cells restores TNFα-induced suppression of AQP2 expression (Lin et al., 2016). In the colon of irritable bowel syndrome rat model, the AQP3 expression was also found to be downregulated by NF-κB (Chao and Zhang, 2017). Therefore, NFκB-mediated inflammatory pathway may play a critical role in the regulation of aquaporin expression in the kidney.

Emerging evidence suggests that there is a strong inflammatory response in the process of HUA (Wu et al., 2021; Yin et al., 2022). The inflammation and oxidative stress are the most primary causative factors in HUA-induced kidney injure (Liu et al., 2021). Studies have shown that UA induces the expression of pro-inflammatory cytokines and chemokines in the kidney, such as interleukin-1β (IL-1β), tumor necrosis factor (TNF)-α, and IL-8 (Chen et al., 2019). Accumulating evidence has indicated that the NF-κB signaling pathway is activated by UA. It has been reported that monosodium urate (MSU) crystals can activate the NF-κB signaling pathway through toll-like receptors 2 (TLR2) and TLR4 (Ru et al., 2005), while soluble UA activates NF-κB by activating mitogen-activated protein kinase (MAPK) (Joosten et al., 2020).

Therefore, we speculate that UA may regulate the expression of AQPs through inflammatory pathways, resulting in impaired renal urine concentration. The purpose of this study was to investigate the effect of HUA on renal AQP expression and its underlying mechanism.

## 2 Materials and methods

### 2.1 Human study

Clinical information and samples from patients and healthy volunteers were obtained after approval by the Ethical Committee on Human Research of the participating hospital and with patient consent. The inclusion criteria for hyperuricemic participants were patients who diagnosed with HUA for the first time in clinic without medication. Basic data was collected including age, gender, height and weight. Body mass index (BMI) was calculated by dividing the weight in kilograms by the square of height in meters. The levels of sUA, serum creatinine (sCr) and blood urea nitrogen (BUN) were measured, and the glomerular filtration rates were estimated. The 24-h urine was collected, and the quantification of 24-h urinary creatinine and uric acid were performed. The above data of hyperuricemic participants were compared with gender- and age-matched normal individuals. None of the participants had other underlying diseases.

### 2.2 Animal study

All animals were purchased from HFK BIOSCIENCE CO.LTD (Beijing, China). The male C57BL/6J mice aged 8-week-old were used in this study. All experiments were reviewed and approved by the Animal Care and Use Review of Dalian Medical University.

### 2.3 Establishment of a mouse HUA model

The C57BL/6 mice were given potassium oxonate (PO, 300 mg/kg) combined with hypoxanthine (HX, 100 mg/kg) suspended in 0.5% sodium carboxymethylcellulose (CMC-Na) by intraperitoneal injection for 7 days.

### 2.4 Chemicals and reagents

PO, HX and UA were purchased from Sigma-Aldrich (St. Louis, MO, USA). Bay11-7,082 was purchased from MCE (Merced, New Jersey, USA). Antibodies against NF-κB and *P*-NF-κB were purchased from Cell Signaling Technology (Danvers, MA, USA). Antibodies against AQP2, AQP3, AQP4 were purchased from Abcam (Cambridge, UK). All secondary antibodies were purchased from ABclonal Technology Co., Ltd (Wuhan, China).

### 2.5 Measurements of sUA, BUN and sCr

Mice in the control group and HUA group were intraperitoneally injected with vehicle or PO + HX for 7 consecutive days. On the sixth day, after intraperitoneal injection the mice were put into the metabolic cages with or without access of water and the 24-hour urine samples were collected. On the seventh day, the mice were taken out of the metabolic cage before intraperitoneal injection. Two hours after the last injection of vehicle or PO + HX, blood and kidney tissues were collected for further analysis. Commercially available kits (C011-2-1, C013-2, C012-2-1) were purchased from Nanjing Jiancheng Bioengineering Institute (Nanjing, China) for the detection of sUA, BUN and sCr levels.

### 2.6 Primary culture of rat inner medullary collecting duct (IMCD) cells

Two 8-week-old male rats were anesthetized with tribromoethanol (25 mg/kg) intraperitoneally, and the kidneys were removed under sterile conditions, and washed three times with ice-cold aseptic PBS buffer. Renal inner medulla samples were digested in a hyperosmotic enzyme solution containing 10 mL DMEM/F12 solution (600 mOsm) supplemented with 120 mM NaCl, 80 mM urea, 20 mg collagenase, and 10 mg hyaluronidase at 37°C for 30 min. The digested cell suspension was centrifuged at 1000rpm for 3min, and the sediment was washed three times with ice-cold DMEM/F12 medium. The IMCD cell pellets were finally resuspended in hyperosmotic DMEM/F12 medium containing 100 IU/mL Penicillin–streptomycin, 10% foetal bovine serum.

### 2.7 Cell viability assay

Cell viability was determined using the Cell Counting Kit-8 assay (Merced, New Jersey, United States). CCK-8 solution was added to the medium of each well of the plate. The cells were cultured for 2 h. Finally, the absorbance was measured at 450 nm using a microplate reader (TECAN, Mannedorf, Switzerland).

### 2.8 Real-time PCR

Total RNA was extracted from mouse kidneys and cultured IMCD cells by using TRIZOL reagent (Vazyme, Nanjing, China), which was then reverse-transcribed to cDNA by using PrimeScript RT reagent kit (Thermo, United States) according to the manufacturer’s instructions. RT-PCR was carried out by using cDNA as template in the PCR reaction with SYBR Green Mix (Vazyme, Nanjing, China). The PCR amplification system was 94°C for 5min, 38 cycles of 94°C for 30s, 65°C for 30s, and 72°C for 30s, with last extension at 72°C for 5min. The relative mRNA expression and fold change of target genes were based on the 2^−ΔΔCT^ method and normalized against GAPDH, which served as the internal reference (Xu et al., 2021). The primers were designed according to the appropriate gene sequences in Pubmed and described in Supplementary Table S1.

### 2.9 Western blot analysis

Primary cultured rat IMCDs or renal medulla tissues were lysed in the Radio Immuno-precipitation Assay (RIPA) buffer containing protease inhibitor cocktail (HY-K0010, MedChemExpress) and phosphatase inhibitors. The lysates were then centrifuged at 12,000 g at 4°C for 15 min, and the supernatants were collected for immunoblotting analysis. The concentration of protein was quantified by the BCA assay kit. Then the total protein was mixed with 6×SDS-PAGE loading buffer, and heated for 10 min. 20 μg cell protein or 60 μg kidney tissues protein were fractionated with 8% or 10% SDS-PAGE and transferred to the NC membrane. The membranes were blocked with 5% bovine serum albumin (BSA) for 1 h on a horizontal rotator at room temperature and incubated with primary antibodies overnight at 4°C. The next day, the membranes were incubated with the secondary antibody for 1 h at room temperature. Finally, the membranes were incubated with ECL reagent, and signals from immunoreactive bands were visualized using a Chemi-luminescent Imaging System (Tanon 5200; Shanghai, China). Densitometric analysis was performed and protein expression level was quantified by Image J software and normalized to the expression of GAPDH.

### 2.10 Histopathology and immunohistochemistry

Kidney tissues were fixed in 4% paraformaldehyde (PFA) for 24 h, embedded in paraffin and then cut into 4 μm sections which were dewaxed and rehydrated for immunohistochemical analysis. The pieces of tissue for the immunohistochemical analysis were immersed in 3% H_2_O_2_ for 8 min to remove endogenous peroxidases. The sections were incubated with the primary antibody overnight at 4°C in a humid environment. In the following day, samples were stained with diaminobenzidine (DAB) kit and finally counterstained with hematoxylin followed by dehydration.

### 2.11 Immunofluorescence assay

Cultured IMCD cells were fixed in 4% PFA for immunofluorescence. After the PFA was removed, the cells were washed twice with ice-cold PBS and incubated with 0.1% BSA at 25°C for 30 min. Then the cells were soaked in primary antibodies at 4°C overnight, then incubated with Fluorescein Isothiocyanate (FITC)-labelled Alexa Fluor‐488 conjugated secondary antibody for 1 h. The cell nucleus was counterstained using DAPI (Beyotime, Shanghai, China). Fluorescence staining was observed under a microscope.

### 2.12 Luciferase activity assay

The HEK293T cells were seeded in 12-well culture plates to reach 80%–90% confluency in DMEM basic medium containing 10% FBS. The cells were co- transfected with the indicated mouse AQP2, AQP3 or AQP4 gene promoter-driven luciferase reporter vector (a kind gift from Dr. R. Qiao at Dalian Medical University) and renilla luciferase, with a mouse NF-κB expression plasmid or pcDNA. After incubation for 24h, the cells were harvested and detected using Dual Luciferase Reporter Assay (Promega, Madison, WI, United States). The firefly luciferase signal was normalized to that of the renilla luciferase signal.

### 2.13 Chromatin immunoprecipitation assay(ChIP)

The chromatin was prepared according to the manufacturer’s protocol of ChIP-IT Express Chromatin Immunoprecipitation Kit (Active Motif, Carlsbad, CA, United SttaesA). Following enzymatic digestion, the digested chromatin was subjected to the ChIP assay by incubating with magnetic beads and antibodies (IgG or NF-κB antibody) overnight. Subsequently, the complex was purified to isolate DNA. The sequences of the primers used are as follows:

AQP2: 5′-AGGTCACTGGACACAGCCTC-3′(F),

5′-ATC ACCCCATCTTAGCTTTCACA-3′(R);

AQP3: 5′-TACATCGATGGGAGTGGGAAA-3′(F),

5′-GGATACCTGGGCTTTCTCCTT-3′(R);

AQP4: 5′-CCCAGTGCTTAGGGGAGTTCT-3′(F), 5′-GCCTCTGGCCCTTAAAGTCATT-3′(R).

### 2.14 Electrophoretic mobility shift assay (EMSA)

Nuclear protein isolated from the HEK293T cells transfected with a mouse NF-κB expression plasmid or pcDNA were extracted using the Nuclear Extract Kit (Thermo Scientific, Massachusetts, United States). The unlabeled probes (Supplementary Table S2) containing the potential NF-κB binding site were synthesized from Tsingke (Beijing, China). The probes were biotin end-labeled using the Biotin 3′End DNA Labeling Kit (Thermo Scientific, Massachusetts, United States). The labeled and unlabeled probes were annealed to double-stranded probe DNA. EMSA assays were carried out using the LightShift Chemiluminescent EMSA kit (Thermo Scientific, Massachusetts, United States).

### 2.15 Statistical analysis

SPSS 19.0 was used for statistical analysis. All data were presented as the means ± SD and analyzed by ANOVA for multiple comparisons or Student’s t-test for comparison between two groups. Statistical significance was taken as *P* < 0.05. The figures were drawn using GraphPad prism 9.0.

## 3 Results

### 3.1 Patients with HUA exhibit increased 24-hour urine output.

**FIGURE 1:**
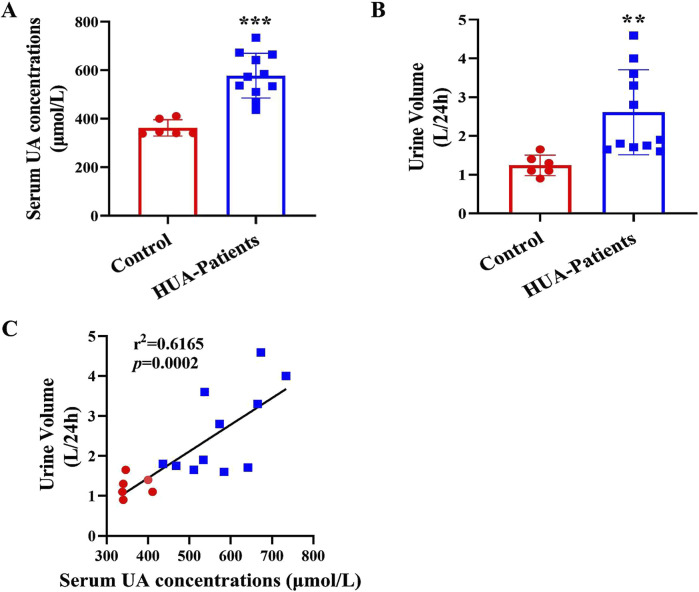
Increased urine output in patients with HUA. **(A)** Serum uric acid (sUA) concentrations in healthy individuals and patients with HUA. **(B)** 24-h urine volume of human participants. **(C)** The correlation analysis of 24-h urine volume and sUA concentrations. Results are presented as means ± SD, ^**^ *p* < 0.01 and ^***^ *p* < 0.001 compared with the healthy control group, n = 6–11.

To determine whether patients with HUA have polyuria, we enrolled 11 male HUA patients and 6 gender- and age-matched healthy volunteers. The clinical characteristics and renal function evaluation of the participants were shown in Supplementary Table S3. Compared with the control group, the serum uric acid (sUA) concentrations and 24-h urine volume of patients with HUA were significantly increased (Figures 1A, B). In addition, 24-h urine volume was positively correlated with sUA concentrations (Figure 1C). These results suggest that patients with HUA exhibit a polyuria phenotype and the urine volume is positively correlated with the sUA concentrations in human.

### 3.2 Mice with HUA exhibit a polyuria phenotype

**FIGURE 2:**
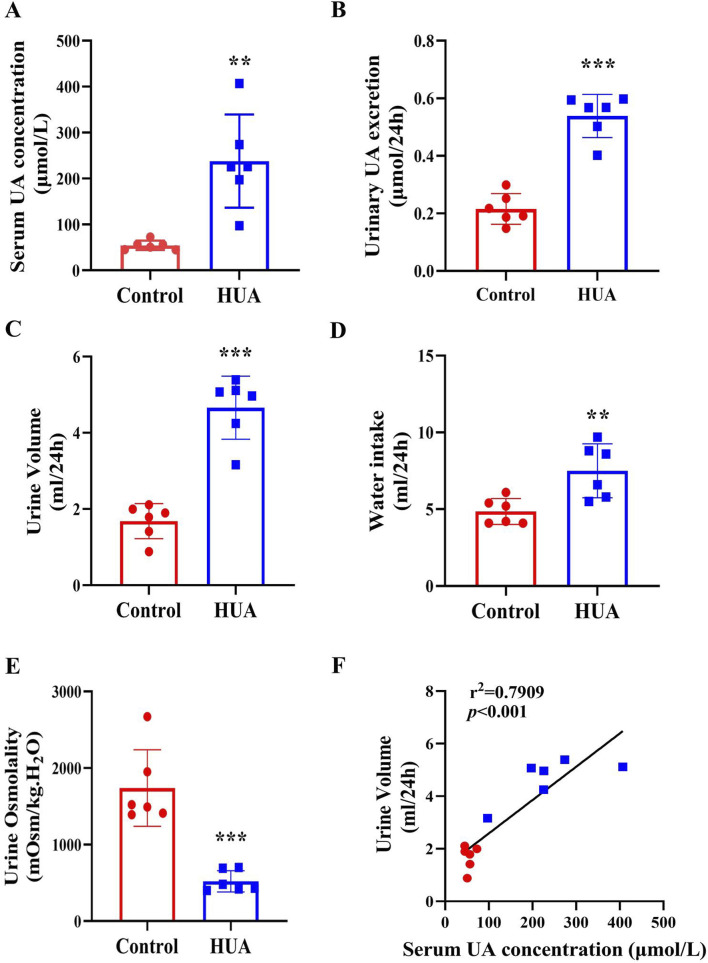
Mice with HUA develops a polyuria phenotype. **(A)** sUA concentrations of mice. **(B)** 24-h urinary UA excretion of mice. **(C)** 24-h urine volume of mice. **(D)** 24-h water intake of mice. **(E)** Urine osmolality of mice. **(F)** The correlation analysis of 24-h urine volume and sUA concentrations. Results are presented as means ± SD, ^**^ *p* < 0.01 and ^***^ *p* < 0.001 compared with the control group, n = 6.

To determine whether HUA also causes polyuria in rodents, 8-week-old male C57BL/6J mice were injected with PO + HX for 7 days to create a model for HUA. Mice were housed in metabolic cages for 24 h with free access to food and water. Similar to hyperuricemic patients, mice with HUA also exhibited a significant increase in sUA concentrations and urine UA excretion (Figures 2A, B), accompanied by a marked increase in 24-h urine volume and water intake and a significant decrease in urine osmolality (Figures 2C–E). Consistent with the findings in HUA patients, there is a positive correlation between sUA concentrations and 24-h urine volume in mice (Figure 2F). In order to exclude the effect of the drugs used in creating UA model on 24-h urine volume, we treated mice with PO or HX alone and found that neither PO or HX affected sUA concentrations (Supplementary Figure S1A), 24-h urine volume (Supplementary Figure S1B) and urine osmolality (Supplementary Figure S1C). Therefore, mice with HUA develops a polyuria phenotype in an hyperuricemia-dependent manner.

### 3.3 The expression of AQP2, AQP3 and AQP4 is decreased in the kidneys of mice with HUA

**FIGURE 3:**
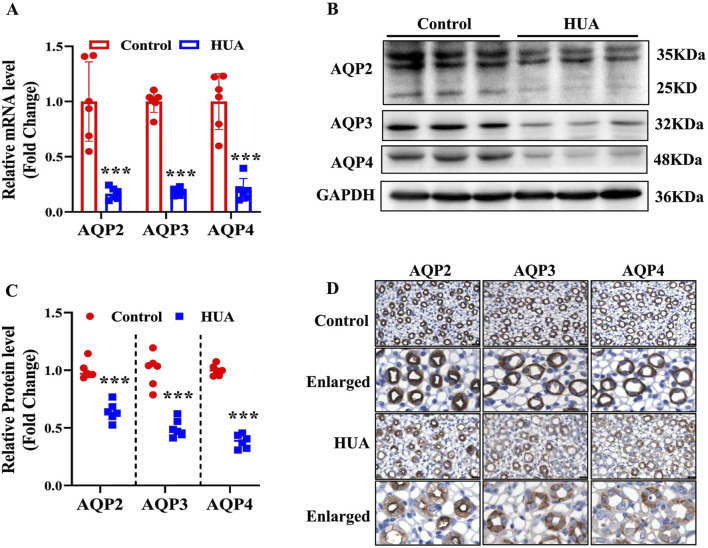
Reduced expression of AQP2, AQP3 and AQP4 in the kidneys of mice with HUA. **(A)** Real-time PCR assay showing the mRNA levels of AQP2, AQP3 and AQP4. **(B)** Representative western blot analysis demonstrating the protein levels of AQP2, AQP3 and AQP4. **(C)** Quantitative analysis of AQP2, AQP3 and AQP4 protein levels in the kidneys of control and HUA mice. **(D)** The representative images of immunohistochemistry studies showing reduced protein expressions of AQP2, AQP3 and AQP4 in mice with HUA. Bar = 20 μm. Results are presented as means ± SD, ^***^ *p* < 0.001 compared with the control group, n = 6.

Urine concentration is important for maintaining body water homeostasis, and is dependent on appropriate expression of AQP2, AQP3 and AQP4 located in the principal cells of renal collecting ducts. Since hyperuricemic mice show impaired urinary concentrating ability, we determined the expression levels of AQP2, AQP3 and AQP4 in the kidneys. The results of real-time PCR and western blot assays revealed that both mRNA and protein expression of AQP2, AQP3 and AQP4 were decreased in the HUA mice compared with control mice (Figures 3A–C). The results of immunohistochemistry study further showed that the protein expression of AQP2, AQP3 and AQP4 in renal collecting ducts were decreased in the HUA group (Figure 3D). Together, these results demonstrate that collecting duct expression of AQP2, AQP3 and AQP4 at both mRNA and protein levels is reduced in mice with HUA.

### 3.4 Mice with HUA produce more urine than control mice under water deprivation

**FIGURE 4:**
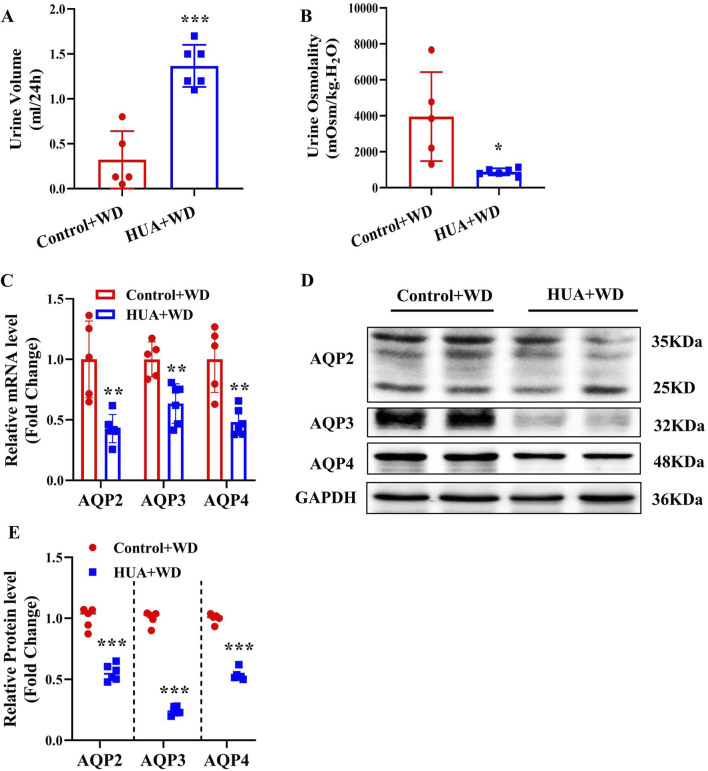
Mice with HUA produce more urine than control mice under water deprivation. **(A)** 24-h urine volume of mice with water restriction for 24 h. **(B)** Urine osmolality of mice. **(C)** Real-time PCR assay showing the mRNA levels of AQP2, AQP3 and AQP4. **(D)** Representative western blot assay showing the protein levels of AQP2, AQP3 and AQP4. **(E)** Quantitative analysis of AQP2, AQP3 and AQP4 expressions in the kidneys of control and HUA mice. Results are presented as means ± SD, ^*^ *p* < 0.05, ^**^ *p* < 0.01 and ^***^ *p* < 0.001 compared with the control group, n = 5–6. WD, water deprivation.

In order to rule out the possibility that increased urine output in the HUA mice is caused by more water intake, both control and HUA mice were subjected to water deprivation(WD) for 24 h. Under water restriction condition, mice with HUA still showed significantly increased 24-h urine volume and decreased urine osmolality compared with the control group (Figures 4A, B). Furthermore, the expression of AQP2, AQP3 and AQP4 at both mRNA and protein levels in the kidneys was significantly lower in the HUA mice (Figures 4C–E). These results suggest that the polyuria phenotype in the HUA mice is mainly due to impaired urine concentration rather than increased water intake.

### 3.5 Treatment of primary cultured IMCDs with UA reduces the expression of AQP2, AQP3 and AQP4

**FIGURE 5:**
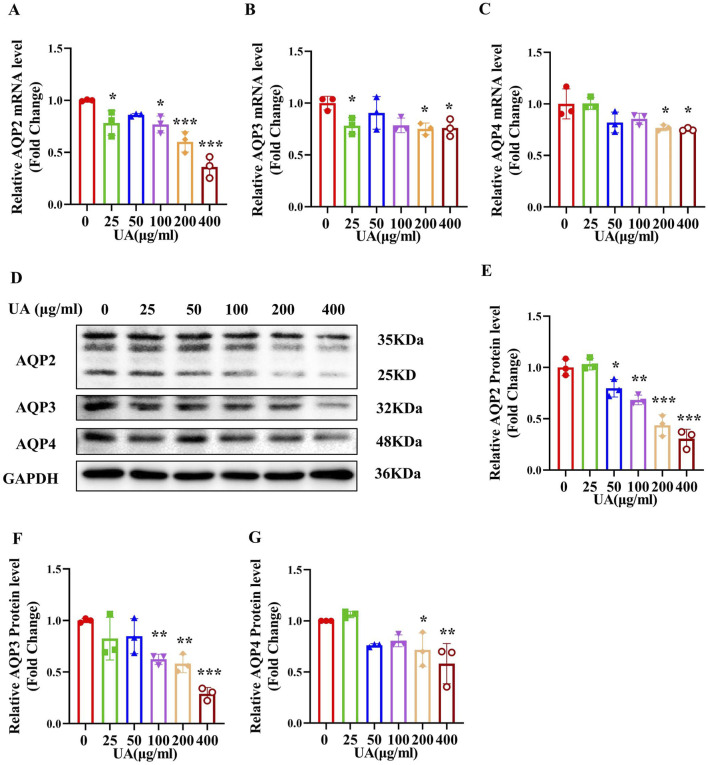
Treatment of primary cultured IMCDs with UA reduces the expression of AQP2, AQP3 and AQP4. IMCDs were treated with UA at various concentrations for 24 h **(A–C)** Real-time PCR assay showing the mRNA levels of AQP2, AQP3 and AQP4. **(D)** Western blot analysis demonstrating the protein levels of AQP2, AQP3 and AQP4. **(E–G)** Quantitative analysis of AQP2, AQP3 and AQP4 protein expression in the IMCDs. Results are presented as means ± SD, ^*^ *p* < 0.05, ^**^ *p* < 0.01 and ^***^ *p* < 0.001 compared with the mice without UA treatment (0 μg/mL), n = 3.

In order to determine the direct effect of UA on the expression of AQP2, AQP3 and AQP4, we treated primary cultured IMCD cells with various concentrations (25, 50, 100, 200 and 400 μg/mL) of UA for 24 h. Real-time PCR and western blot analysis revealed that UA treatment resulted in a significant decrease in the levels of AQP2, AQP3 and AQP4 expression at both mRNA (Figures 5A–C) and protein (Figures 5D–G). Exposure of the cells to UA at the indicated doses had little effect on cell morphology (Supplementary Figure S2A) and cell viability (Supplementary Figure S2B) and did not cause cell apoptosis (Supplementary Figure S2C). These results indicate that uric acid can directly reduce the expression of AQP2, AQP3 and AQP4 in the IMCD cells.

### 3.6 NF-κB is activated in HUA mouse kidneys and UA-treated IMCD cells

**FIGURE 6:**
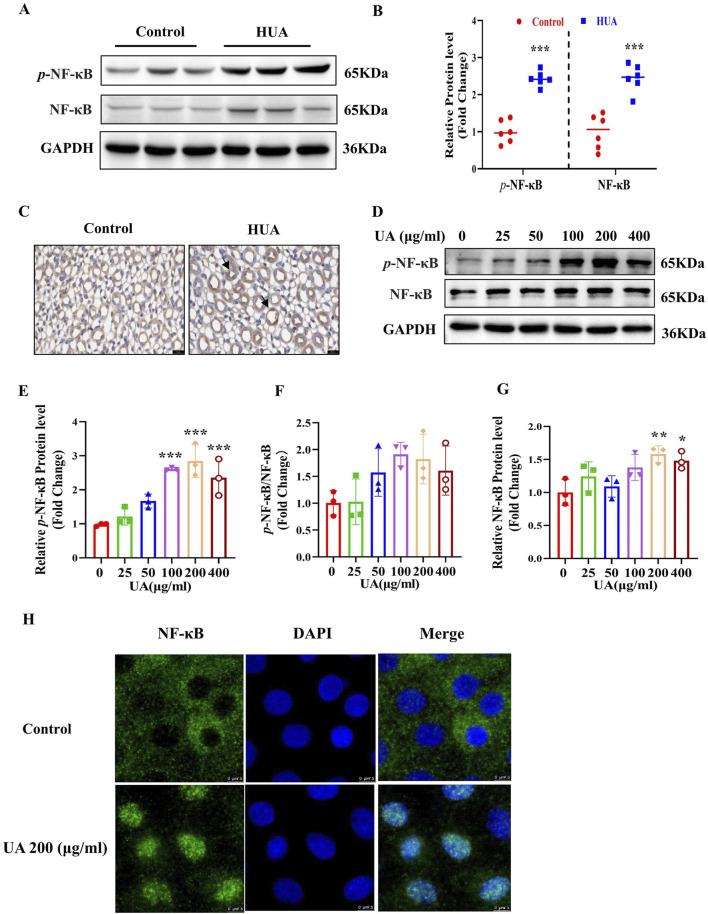
NF-κB is activates in HUA mouse kidneys and UA-treated IMCD cells. **(A)** Representative western blot assay showing the protein levels of *p*-NF-κB and NF-κB in the kidneys of control and HUA mice. **(B)** Quantitative analysis of western blot on *p*-NF-κB and NF-κB in the kidneys of control and HUA mice. **(C)** Immunohistochemistry study demonstrating increased expression and nuclear translocation of NF-κB in mouse collecting ducts. The black arrows represent nuclear localization of NF-κB. Bar = 20 μm. **(D)** Western blot analysis showing the protein levels of *p*-NF-κB and NF-κB in cultured IMCD cells treated with UA at various concentrations for 24 h. **(E–G)** Quantitative analysis of *p*-NF-κB and NF-κB protein levels in Figure D, respectively. **(H)** Immunofluorescence experiment demonstrating UA-induced nuclear translocation of NF-κB in cultured IMCD cells. Bar = 7.5 μm. Results are presented as means ± SD, ^*^ *p* < 0.05, ^**^ *p* < 0.01 and ^***^ *p* < 0.001 compared with the control group (0 μg/mL), n = 3.

As previously reported, uric acid crystals can directly interact with Toll-like receptors (TLRs), initiating signal transduction pathways that ultimately activate NF-κB (Du et al., 2024) and NF-κB can reduce AQP2 gene transcription (Hasler et al., 2009). We speculate that UA may regulate the expression of AQPs through NF-κB. We found that mice with HUA exhibited a significantly higher levels of *p*-NF-κB and NF-κB in the kidneys compared with the control group as assessed by western blot analysis (Figures 6A, B). Immunohistochemistry study further showed an increased expression and nuclear translocation of NF-κB in the collecting ducts of mice with HUA (Figure 6C). Consistent with the *in vivo* findings, *in vitro* study also revealed that UA treatment for 24 h markedly increased the protein expression of *p*-NF-κB and NF-κB in cultured IMCD cells (Figures 6D–G). Immunofluorescence experiment further showed that UA promoted NF-κB translocation to the nuclei of IMCD cells (Figure 6H). Collectively, both *in vivo* and *in vitro* studies demonstrate that UA may induce the activation of NF-κB in renal collecting ducts.

### 3.7 Inhibition of NF-κB abolishes UA-induced downregulation of AQP2, AQP3 and AQP4 in cultured IMCDs

**FIGURE 7:**
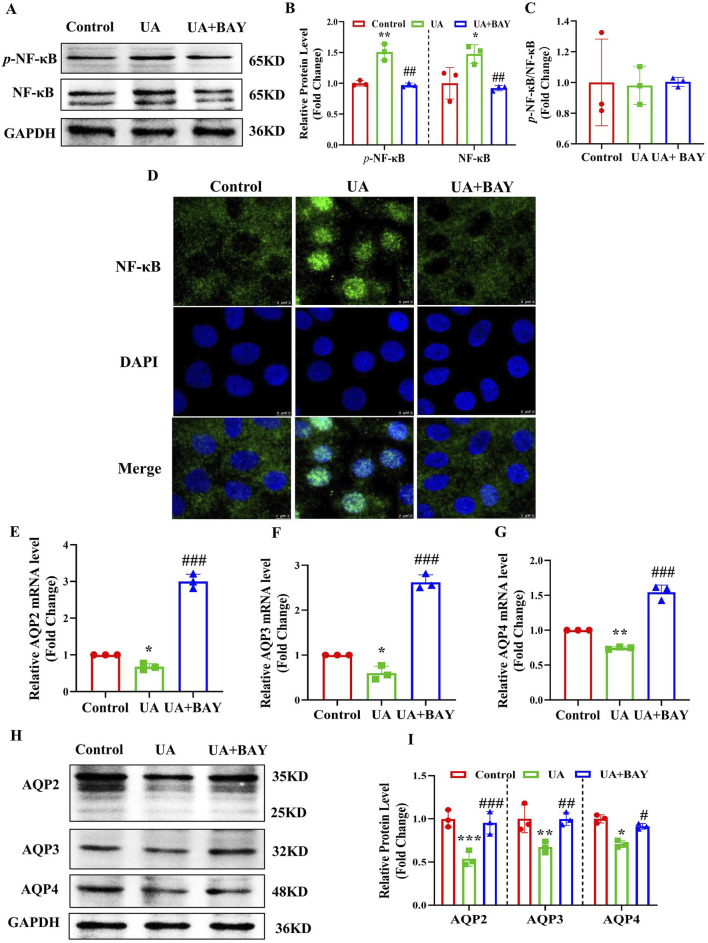
Inhibition of NF-κB abolishes UA-induced downregulation of AQP2, AQP3 and AQP4 in cultured IMCDs. IMCD cells were treated with UA (200 μg/mL) in the presence or absence of the NF-κB inhibitor BAY11-7082 (2 μM) for 24 h. **(A)** Western blot assay showing the protein levels of *p*-NF-κB and NF-κB. **(B, C)** Quantitative analysis of protein expressions in Figure A. **(D)** Immunofluorescence study demonstrating the nuclear translocation of NF-κB. **(E–G)** Real-time PCR assay showing the mRNA levels of AQP2, AQP3 and AQP4. **(H)** Western blot analysis showing the protein levels of AQP2, AQP3 and AQP4. **(I)** Quantitative analysis of protein expressions in Figure H. Results are presented as means ± SD, ^*^ *p* < 0.05, ^**^ *p* < 0.01 and ^***^ *p* < 0.001 compared with the control group; ^#^ *p* < 0.05, ^##^p < 0.01 and ^###^p < 0.001 compared with the UA group, n = 3.

To clarify whether UA reduces the expressions of AQP2, AQP3 and AQP4 through the NF-κB signaling, we treat IMCD cells with UA (200 μg/mL) in the presence or absence of the NF-κB inhibitor BAY11-7082 (2 μM) for 24 h. Western blot analysis revealed that BAY11-7082 significantly attenuated UA-induced protein expression of *p*-NF-κB and NF-κB (Figures 7A–C). In addition, immunofluorescence study showed that BAY11-7082 blocked UA-induced nuclear translocation of NF-κB in the IMCD cells (Figure 7D). Real-time PCR and western blot analysis further revealed that BAY11-7082 significantly reversed UA-induced downregulation of AQP2, AQP3 and AQP4 at both mRNA (Figures 7E–G) and protein (Figures 7H, I) levels. These results demonstrate that NF-κB mediates the UA-induced downregulation of AQP2, AQP3 and AQP4 in the IMCDs.

### 3.8 NF-κB binds to the promoter regions of mouse AQP2, AQP3 and AQP4 gene to suppress their transcription

**FIGURE 8:**
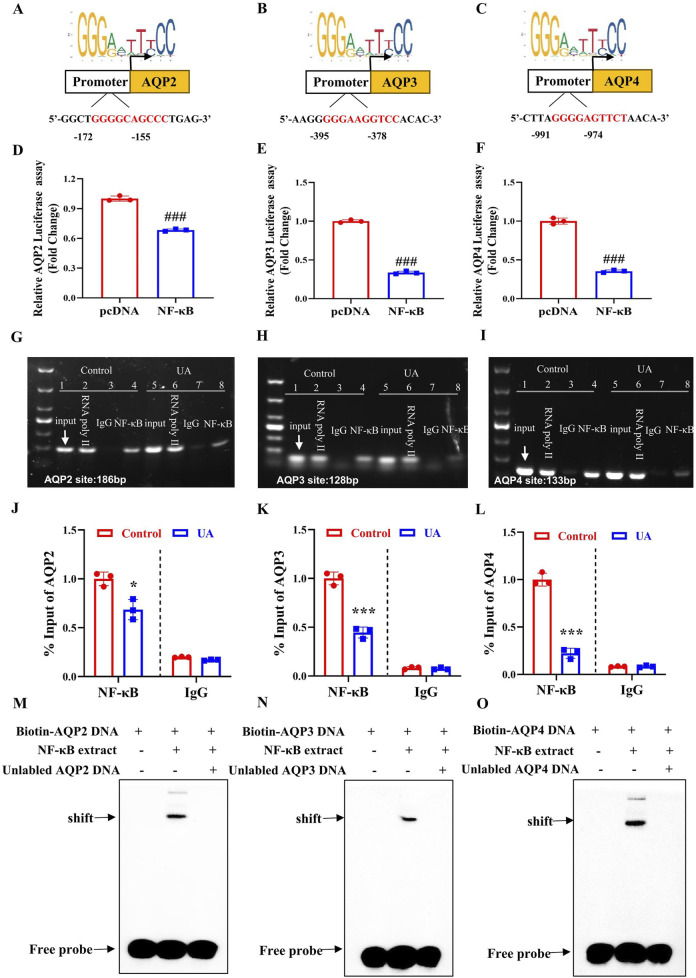
NF-κB binds to the promoter regions of mouse AQP2, AQP3 and AQP4 genes to suppress their transcription. **(A–C)** The JASPAR CORE database was used to predict the putative NF-κB binding element in the promoter regions of mouse AQP2, AQP3, and AQP4 genes. **(D–F)** Luciferase reporter activity assay showing the suppressive effect of NF-κB expression on the transcription activity of AQP2, AQP3 and AQP4 gene promoter, respectively. ^###^ *p* < 0.001 compared with the pcDNA group, n = 3. **(G–I)** ChIP assay demonstrating the binding of NF-κB to the AQP2, AQP3 and AQP4 promoter region. **(J–L)** Quantification of the ChIP analysis shown in Figure G–I. **(M–O)** EMSA assay demonstrating direct binding of NF-κB to the predicted NF-κB-binding site located in the promoter region of AQP2, AQP3 or AQP4 gene. The position of the shifted complex and free probes are indicated by arrows. Results are presented as means ± SD, ^*^ *p* < 0.05, ^***^ *p* < 0.001 compared with the control group, n = 3.

To determine the mechanism by which NF-κB suppresses AQP2, AQP3 and AQP4 expression, we analyzed the promoter sequences of mouse AQP2, AQP3 and AQP4 genes using the JASPAR CORE database and found all genes contain a potential NF-κB-binding site, which were located between −172 bp and −155 bp upstream from the transcription start site (TSS) of AQP2 gene (Figure 8A), between −395 bp and −378 bp upstream from the TSS of AQP3 gene (Figure 8B), and between −991 bp and −947 bp upstream from the TSS of AQP4 gene (Figure 8C), respectively. We then transfected a luciferase reporter driven by mouse AQP2, AQP3 or AQP4 gene promoter containing the potential NF-κB-binding site into 293T cells with or without the NF-κB-expressing vector. The result showed that NF-κB overexpression significantly decreased the transcription activity of mouse AQP2, AQP3 and AQP4 gene promoter (Figures 8D–F). To verify whether NF-κB could directly bind to the predicted promoter regions of AQP2, AQP3 or AQP4 gene, ChIP assay was performed. In the basal condition, we confirmed the binding of NF-κB to the predicted sequence located in the AQP2, AQP3 or AQP4 promoter region, all of which was decreased by UA treatment (Figures 8G–L). Furthermore, we utilized EMSA to confirm the binding of NF-κB to the predicted promoter sequence of the AQP2, AQP3 and AQP4 gene, respectively (Figures 8M–O). Collectively, these findings demonstrate that NF-κB binds to the promoter regions of mouse AQP2, AQP3 and AQP4 genes to suppress their transcription.

## 4 Discussion

Hyperuricemia (HUA) represents a risk factor for many diseases including hypertension and chronic kidney disease. Clinical observations suggest that patients with HUA appear to have increased urine output (Johnson et al., 2018b; Li et al., 2019). The present study provided direct evidence that both patients and mice with HUA exhibit a polyuria phenotype. We found that the expression of AQP2, AQP3 and AQP4 was significantly reduced in the kidneys of HUA mice and UA-exposed IMCDs. We further observed an increased expression of NF-κB in the renal collecting ducts of mice with HUA and in the IMCD cells treated with UA. Blockade of NF-κB dramatically attenuated UA-suppressed the expression of AQP2, AQP3 and AQP4 in cultured IMCDs. Furthermore, the luciferase reporter, CHIP and EMSA assays all showed that NF-κB can suppress the expression of AQP2, AQP3 and AQP4 at the transcriptional level. Collectively, our findings demonstrate that UA reduces the expression of AQP2, AQP3 and AQP4 in an NFκB-dependent manner, which contributes to the polyuria phenotype in subjects with HUA.

In the present study, we determined the 24-h urine volume in newly diagnosed hyperuricemic patients without medication and found a significantly increased urine output compared to the age- and gender-matched healthy individuals. Similarly, we established a mouse model of HUA and observed a marked increase in 24-h urine excretion under both basal and dehydrated conditions. In addition, in both patients and mice, the urine volume is closely correlated with the levels of serum UA concentrations. These findings indicate that HUA can indeed cause polyuria, which is likely caused by HUA and is independent of increased water intake. At present, the clinical significance of HUA-induced polyuria is not clear. Due to increased urinary UA excretion observed in the HUA mice, it is possible that increased urine output may help eliminate uric acid to reduce serum UA concentration.

Water content in human body maintains a dynamic balance through water intake and excretion. The kidney is a central organ in water homeostasis by producing urine to eliminate excessive water. In the process of urine concentration and dilution, renal AQPs are the key proteins that determine the final urine volume, especially AQP2, AQP3 and AQP4 in the principal cells of renal collecting ducts (Su et al., 2020). AQP2 is localized at the apical membrane, while AQP3 and AQP4 are expressed at the basolateral membrane of renal collecting ducts. Increasing evidence demonstrates that AQP2 expression is under the control of many factors including arginine vasopressin (AVP), prostaglandin E2 receptor 4 (EP4), farnesoid X receptor (FXR) and NF-κB (Kharin and Klussmann, 2024; Deen et al., 2021; Xu et al., 2018). In the present study, we found that expression of AQP2, AQP3, and AQP4 at both mRNA and protein levels was significantly reduced in the kidneys of HUA mice and UA-exposed IMCD cells. These findings indicate that UA can directly suppress AQP2, AQP3 and AQP4 expression in renal collecting duct cells, contributing to the pathogenesis of polyuria in mice with HUA.

UA plays an important antioxidant role in the body at physiological concentrations. However, when the sUA level exceeds the physiological range, UA induces inflammatory response (Wen et al., 2021; Jo et al., 2015). It has been previously reported that UA can activate the NF-κB pathway via TLR6 in cardiomyocytes (Zhang et al., 2020). Studies also showed that activation of the NF-κB inflammatory pathway in renal diabetes insipidus induced by lithium chloride is associated with a decrease in AQP2 expression (Sung et al., 2019), and NF-κB can bind to mouse AQP2 gene promoter region to inhibit transcription of AQP2 (Hasler et al., 2008). To identify the underlying mechanism by which UA suppresses the expression of AQP2, AQP3 and AQP4, we measured the expression of NF-κB in the kidneys of HUA mice and found the expression and nuclear translocation of NF-κB were markedly increased in the medullary collecting duct cells. Similarly, UA treatment also significantly induced the protein expression and nuclear localization of NF-κB in cultured collecting duct cells. Since NF-κB has been previously reported to be capable of suppressing AQP2 expression (Hasler et al., 2008), these findings suggest that UA reduce AQP2, AQP3, and AQP4 expression via inducing NF-κB expression and activity. In support, inhibition of NF-κB markedly abolished the suppressive effect of UA on the expression of AQP2, AQP3, and AQP4 in the IMCDs. Together, these results demonstrate that UA suppresses the expression of AQP2, AQP3, and AQP4 in the IMCDs in an NF-κB-dependent manner.

By analyzing the sequences of mouse AQP2, AQP3, and AQP4 genes, we identified a potential NF-κB binding site located in the promoter region of each gene. Subsequent studies using Luciferase reporter, CHIP and EMSA techniques all confirmed that NF-κB can bind to the predicted NF-κB binding site in each gene promoter, resulting in the suppression of the transcription of AQP2, AQP3, and AQP4 gene. Collectively, these findings demonstrate that NF-κB could directly bind to the promoter regions of mouse AQP2, AQP3 and AQP4 genes to downregulate their expression.

In conclusion, we report that HUA can damage urine concentration capacity leading to a polyuria phenotype via down-regulating AQP2, AQP3 and AQP4 expression in the kidney. UA-induced NF-κB expression and activation is responsible for suppressed expression of AQP2, AQP3 and AQP4 in renal collecting ducts.

## Data Availability

The original contributions presented in the study are included in the article/Supplementary Material, further inquiries can be directed to the corresponding authors.
