# Supplementary material for: Uric acid reduces the expression of aquaporins in renal collecting ducts to increase urine output in hyperuricemia
Source: Front Physiol. 2025 Apr 9;16:1504328. doi: 10.3389/fphys.2025.1504328 (PMC12014756; doi:10.3389/fphys.2025.1504328)

Supplemental Information

Supplemental Figure Legends

Fig.S1. Effect of the drugs used in creating UA model on 24-h urine volume in mice.

(A) sUA concentrations of mice treated with potassium oxonate (PO, 300mg/kg), hypoxanthine (HX, 100mg/kg) or both. (B) 24-h urine volume of mice. (C) Urine osmolality of mice. Results are presented as means ± SD, **p*<0.05, ***p*<0.01 and ****p*<0.001 compared with the control group, n=4-5.

Fig.S2. Effect of UA treatment on the morphology, viability and apoptosis of IMCD cells.

(A) Light microscopic images of cell morphology. (B) CCK8 assay showing the cell viability. (C) TUNEL assay was used to test the apoptosis of cells. Bar=25μm.

Supplemental Tables

Table S1. Primers for real-time PCR.

GENE	Species	Forward primer (5'-3')	Reverse primer (5'-3')
AQP2	Mouse	TTGCCATGTCTCCTTCCTTC	GGTCAGGAAGAGCTCCACAG
AQP3	Mouse	CCCTCTGGACACTTGGACAT	GTTGACGGCATAGCCAGAAT
AQP4	Mouse	TTGCTTTGGACTCAGCATTG	GGGAGGTGTGACCAGGTAGA
AQP2	Rat	ACCTGGCTGTCAATGCTCTC	CAGCTGCATGGTCAGGAAGA
AQP3	Rat	AAGCCAAGTTGATGGTGAGG	GGGGACCCTCATCCTTGT
AQP4	Rat	TATCCAGTGGTTTTCCCAGT	GCAATTGGACATTTGTTTGC
GAPDH	Mouse	TCAGGAGAGTGTTTCCTCGT	GAGGTCAATGAAGGGGTCGT
GAPDH	Rat	GTCGGTGTGAACGGATTTGG	TCCCGTTGATGACCAGCTTC

Table S2. Primers for EMSA.

GENE	Forward primer(5'-3')	Reverse primer(5'-3')
AQP2	CATTGTGGGGGCTGGGGCAGCCCTGAGGCA	TGCCTCAGGGCTGCCCCAGCCCCCACAATG
AQP3	CTCAGCCAAGGGGGAAGGTCCACAAGGAGA	TCTCCTTGTGGACCTTCCCCCTTGGCTGAG
AQP4	AGTGCTTAGGGGAGTTCTAACACTCAGTAA	TTACTGAGTGTTAGAACTCCCCTAAGCACT

Table S3. Clinical characteristics and renal function evaluation of human participants.

Characteristics	Control (n=6)	HUA-Patients (n=11)	*P* value
Age (years)	32.33± 12.21	29.91±10.55	0.674
Gender (male%)	6(100%)	11(100%)	1
BMI (kg/m^2)	24.34±4.09	28.817±3.50	0.031
eGFR (ml/(min*1.73 m^2))	117.84±8.44	112.12±14.25	0.401
Serum creatinine (μmol/L)	76.52±3.97	78.12±9.42	0.7
Urinary creatinine (mmol/24h)	19.56±3.35	15.87±3.65	0.076
Urinary uric acid (mmol/24h)	4.76±2.24	4.77±2.31	0.993

BMI, body mass index; eGFR, estimated glomerular filtration rate.

Supplemental Figures

Fig.S1

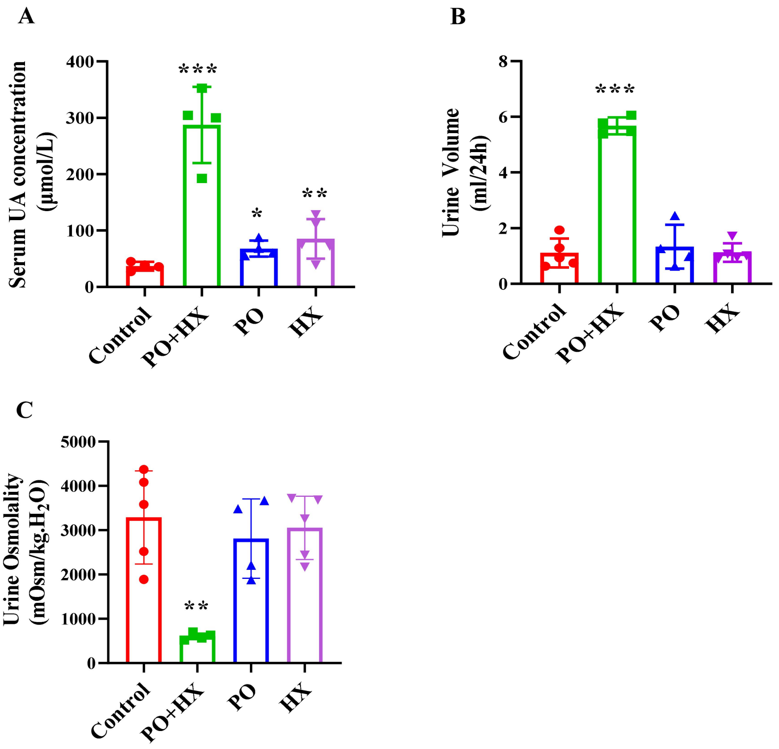

Fig.S2

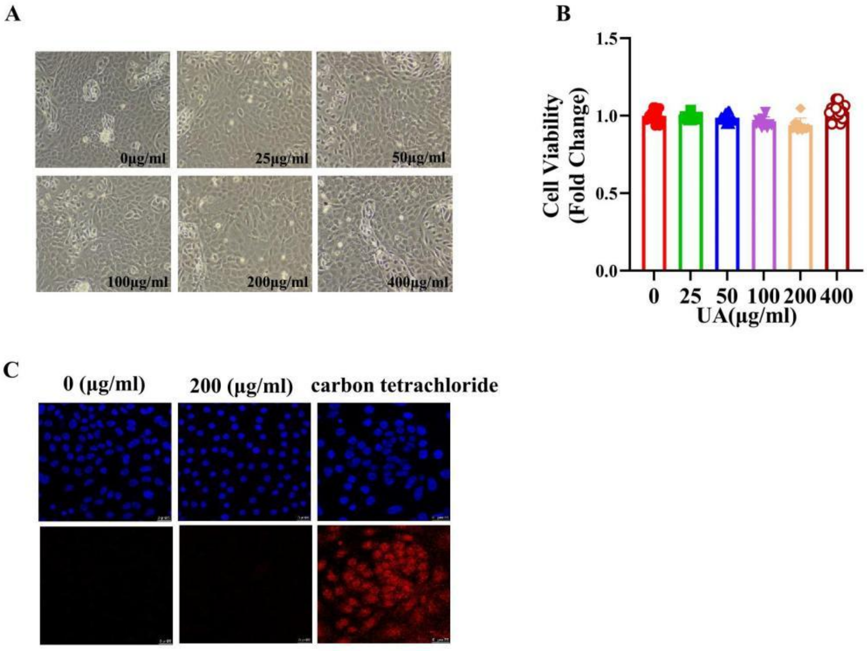

Supplement: Supplementary file 1 [file DataSheet1.pdf]
